# Retraction: Akt2 Regulates the Differentiation and Function of NKT17 Cells *via* FoxO-1-ICOS Axis

**DOI:** 10.3389/fimmu.2021.686304

**Published:** 2021-03-31

**Authors:** Frontiers Editorial Office

The journal and Chief Editors retract the 12 September 2018 article cited above.

Following the publication of the article, image duplication concerns were identified in the published article, specifically in Figure 3J. The authors were unable to provide a satisfactory explanation for this occurrence despite issuing a Correction article. As a result, the data and conclusions of the article have been deemed unreliable. The article is therefore being retracted.

The authors concur with the retraction and sincerely regret any inconvenience this may have caused to the reviewers, editors and readers of Frontiers in Immunology.

